# Genistein Induces Apoptosis and Inhibits Proliferation of HT29 Colon Cancer Cells

**Published:** 2016-08-30

**Authors:** Gholamreza Shafiee, Massoud Saidijam, Heidar Tavilani, Neda Ghasemkhani, Iraj Khodadadi

**Affiliations:** 1 *Department of Biochemistry, Faculty of Medicine, Hamadan University of Medical Sciences, Hamadan, Iran.*; 2 *Department of Molecular Medicine and Human Genetics, Faculty of Medicine, Hamadan University of Medical Sciences, Hamadan, Iran.*

**Keywords:** Apoptosis, caspase-3, colonic neoplasms, genistein, p38 mitogen-activated protein kinase

## Abstract

Soybean isoflavone genistein has multiple anticancer properties and its pro-apoptotic and anti-proliferative effects have been studied in different cancer cells. However, the mechanisms of action of genistein and its molecular targets on human colon cells have not been fully elucidated. Therefore, caspase-3 and p38 mitogen-activated protein kinase (p38 MAPK) as the main therapeutic targets were investigated in this study at both gene expression and protein levels in HT29 colon cancer cells. The caspase-3 and *p38 MAPK *gene expression levels were examined by real time PCR whereas flow cytometry technique was performed to determine their intracellular protein levels. The caspase-3 enzyme activity was obtained by colorimetric method while the gelatinase activity of matrix metalloproteinase-2 (MMP2) was determined by zymography. In addition, MTT test, wound healing assay and clonogenic assay were carried out to determine the effect of genistein on HT29 cell viability, migration, and proliferation, respectively. Genistein induced apoptotic death in HT29 cells through activation of caspase-3 pathway at the transcriptional, protein, and enzymatic levels. Moreover, genistein inhibited the proliferation of HT29 cells by reducing of both *p38 MAPK* gene expression and its active phosphorylated protein level. Also, we showed that genistein strongly suppressed the metastatic potency of HT29 colon cancer cells via the reduction of MMP2 activity. Based on the results of this study, we conclude that genistein may exhibit its anticancer properties on HT29 colon cancer cells by modulating caspase-3 and p38 MAPK pathway at different transcriptional and protein levels.

Colon cancer is one of the most common tumors and a leading cause of cancer deaths for both men and women worldwide (1). The incidence of colon cancer is much higher in the developed countries such as European countries and United States compared with African and Asian countries (2). Epidemiological studies suggest that the improvement in dietary habits and lifestyle together with proper physical activity are the major concerns in the prevention of colorectal cancer (3). For instance, because of the consumption of soy isoflavone-containing foods, the risk of developing colon cancer has been substantially lower in some Asian countries such as Japan and China compared with western countries (4, 5). Genistein (4,5,7-trihydroxyisoflavone) is an isoflavone which has been identified as phytoestrogen component found in soybean enriched foods (6). Possible mechanisms for the anti proliferative property of genistein mainly include antioxidant effect, an inhibitory effect on tyrosine kinases, cell cycle arrest, blocking of estrogen receptor, induction of differentiation and apoptosis, and modulation of the pathways of signal transduction (6-8). Studies have shown that isoflavone genistein induces apoptosis in different types of cancers through several mechanisms including increasing expression of *BAX* and *P21*, decreasing expression of *BCL-2* and activating of caspase enzymes (8,9). Mitogen-activated protein kinases (MAPKs) including ERK, JNK, and p38 MAPK are serine/threonine protein kinases that have been implicated in many cellular processes such as proliferation, differentiation, and apoptosis. Human MAPK pathway is often dysregulated in cancer cells and thus is considered as potential therapeutic target (10). The p38 MAPK is activated by dual phosphorylation at threonine and tyrosine residues in response to a variety of stimuli such as stress, cytokines, inflammation and drugs (11). Activated p38 MAPK (p-p38 MAPK) regulates the activity of a wide range of protein kinases, transcription factors and some of other proteins that are involved in cell apoptosis, proliferation, differentiation, and tumor suppression (12). The active p38 MAPK on the other hand, is down regulated with dephosphorylation by MAPK phosphatase (MKP) family (11). Genistein has been found to inhibit the molecules in the MAPK pathway. For instance, it has been reported that genistein could block the activation of p38 MAPK by inhibiting the phosphorylation of tyrosine resi-due on p38 MAPK, leading to the inactivation of MAPK pathway (13).

Similar anti-proliferative effect of genistein has also been reported in human HT29 colon cancer cell line by Zhu et al. (14). This was later corroborated by further observations that physiological concentrations of dietary phytochemicals including genistein results in the reduced growth and induced apoptosis of cancer cells (15). Since the HT29 cell line (ATCC Number: HTB-38) is isolated from a primary tumor of human colon tissue and is known as characteristic model of human colon cancer cells which produce carcinoembryonic antigen (CEA) and form well-differentiated adenocarcinoma consistent with colonic primary, grade I, in the present study, we investigated possible apoptotic and anti proliferative effects of genistein on HT29 colon cancer cells via the determination of caspase-3 and *p38 MAPK* gene expression level and their corresponding protein concentration.

## Materials and methods

**Study design**

In the present study, HT29 colon cancer cells were cultured with different concentrations of genistein (Sigma-Aldrich Co, Steinheim, Germany) and the effects of different concentrations of genistein (10, 30, 50, 70 and 90 μM in DMSO) on caspase-3 and *p38-MAPK* gene expression and protein levels were investigated. In addition, potential anti proliferative, pro-apoptotic, anti migration, and anti metastatic properties of genistein were investigated by colony formation assay, MTT assay, wound healing assay, and zymography, respectively.

**Cell culture**

Human HT29 colon cancer cell line was purchased from Pasteur Institute Cell Culture Collection (Tehran, Iran). The cells were cultured in a 75-cm^2^ flask containing RPMI1640 media (Life Technologies Ltd., Paisley-UK), 10% FBS (Life Technologies Ltd, Paisley-UK), 10,000 U/ml of penicillin and 10,000 μg/ml of streptomycin and incubated at 37 C with 5% CO_2_. After the cells reached confluence, the medium was removed and cells were washed with phosphate-buffered saline (PBS) (Life Technologies Ltd, Paisley-UK). Cells were then detached from culture flask by the addition of 0.25% Trypsin-EDTA solution (Life Technologies Ltd, Paisley-UK) and were dispensed into new culture plates for further experiments.

**MTT assay to determine the effect of genistein on HT29 cell viability**

Cell viability was quantified using MTT assay (3- [4,5- dimethylthiazo l-2-yl]-2, 5 diphenyl tetrazolium bromide), as previously described (15, 16). Briefly, 110^4^ cells were seeded into a 37 mm diameter culture plates and incubated for 12, 24, 48, 56, and 72 h with or without 0, 10, 30, 50, 70, or 90 µM solution of genistein. Then, 10 µl of MTT reagent (2 mg/ml MTT in PBS) was added into the culture plates and the blue color of formazan produced by mitochondrial succinate dehydro-genase was measured at 570 nm with a Sunrise^TM^ ELISA plate reader (Tecan Group Ltd, Männedorf-Switzerland). Cell survival was calculated using the mean of triplicate experiments. The inhibitory rates for different concentrations of genistein at each incubation time (12, 24, 48, and 72 h) were calculated using the following equation:

Inhibitory rate (IR) (%) = (1 - OD_treatment_/OD_control_) × 100%

Then, inhibitory rates from the incubation time showing the highest regression (48 h) were used to calculate the IC_50_ via following equation where the Low_conc_ and High_conc_ are the lowest and the highest concentrations of genistein, respectively whereas Low_IR_ and High_IR_ represent the lowest and the highest inhibitory rates:

IC_50_= (50%Low_IR_) / (High_IR_Low_IR_) × (High_conc_ Low_conc_) + Low_conc_

**Wound healing assay to determine the effect of genistein on HT29 cell migration**

The effect of genistein on the migration potency of HT29 cells was examined using the wound healing assay, as previously described (17). Cells were cultured in 24-well culture plates and allowed to reach 90% confluence. A wound track (approximately 5 mm in size) was scored in each well with a crystal microsampler followed by washing the cells with PBS to remove cell debris. The cells were then incubated with 0, 10, 30 or 50 µM genistein for 0, 12, 24 and 48 h and the rehabilitation of scratches was visualized under microscope. After acquisition, images were processed using the ImageJ 1.49 software (http://rsb.info.nih.gov/ij/). The areas of scratches were determined at each time point and the width of the gaps between culture areas were calculated by dividing the scratch area by the length. Finally, data for the width of the gaps were presented as micrometer (µm).

**Colony formation assay to determine the effect of genistein on HT29 cell proliferation**

The effect of genistein on the proliferation of HT29 cells was analyzed by the clonogenic assay (18). Briefly, HT29 cells were cultured on 6-well plates at density of 1×10^3^ cells per well and received control media (RPMI1640 media, 10% FBS, 10,000 U/ml of penicillin and 10,000 μg/ml of streptomycin) or media supplemented with either 0, 10, 30 or 50 μM genistein. Cells were incubated for one week at 37^ o^C with 5% CO_2_ until the cells in control plates have formed colonies that were visible to eye and were of a substantially good size (at least 50 cells per colony).

For fixation and staining, media was removed and the cells were washed twice with PBS. The colonies were fixed with 0.5% crystal violet solution for 45 min at room temperature. The plates were then washed with water and air-dried. Finally, the numbers of colonies containing more than 50 cells were counted by light microscopy and plating efficiency (PE) and surviving fraction (SF) were calculated. Each treatment was performed in triplicate.

PE= (Number of counted colonies/Number of plated cells)× 100

SF = (PE of treated sample/PE of control)× 100

**Zymography to determine the effect of genistein on MMP2 activity of HT29 cells**

Gelatinolytic activity of matrix metallopro-teinase-2 (MMP2) was assessed by zymoghrapy, as previously described with some modifications (19). Briefly, cells were cultured in 25 cm^2^ culture flasks in RPMI medium with %10 FBS. The cells were then incubated with 0, 30, 50 or 70 µM genistein for 48 h and then harvested in conditioned medium. The gelatinolytic activity of MMP2 in the conditioned medium samples was assessed by electrophoresis on a 10% polyacrylamide-sodium dodecyl sulfate gel (PAGE) co polymerized with 2% gelatin as substrate. Gel electrophoresis continued until the marker dye reached the bottom of the gel. After electrophoresis, gels were washed twice by gentle agitation (30 min per wash) in 2% Triton X-100 solution at room temperature to enable enzyme renaturation. After decanting the washing solution, gels were immersed in Tris–HCl buffer, pH 7.4 for 30 min at room temperature and were then placed in fresh developing buffer for 72 h at 37 °C. The gels were washed with deionized water and stained with coomassie brilliant blue G-250 (Merck KGaA, Darmstadt, Germany) in 10% methanol and 10% acetic acid for 1 h at room temperature. The gels were then washed with water and soaked in de staining solution for three days until clear bands were seen indicating the MMP2 activity of gelatin degradation. Gelatinolytic activities were detected as unstained bands against the background of coomassie blue-stained gelatin. To confirm gelatinase activities observed in unstained bands (corresponding to MMP2), the experiment was repeated by immersing the gels in EDTA buffer which chelates Zn^2+^ on the active site of MMPs and inhibits enzyme activity leaving no unstained area on the gel. Gelatinase activity of MMP2 was reconfirmed by comparing the unstained bands to the molecular weight marker (ThermoFisher Scientific, Paisley, UK) which showed a protein with 72 kDa molecular weight, as indicated in UniProtKB online database (http:// www.uniprot.org/ uniprot/ P08253). The gels were scanned by Canon LiDE110 scanner (Canon Inc, Tokyo, Japan) and images were analyzed using the ImageJ 1.49 software (http:// rsb.info.nih.gov/ij/) to quantify proteolitic activity of MMP2

**Real-time PCR for determination of caspase-3 and** ***p38 MAPK*** **gene expression**

To determine the apoptotic and anti-proliferative effects of genistein in HT29 cells, the expression of caspase-3 and *p38 MAPK* genes were measured respectively by real-time PCR. Briefly, the cells were incubated with or without 30, 50, and 70 μM solution of genistein for 48 h in 75 cm^2^ culture flasks. Total RNA was extracted using RNXplus reagent (Bioscience, Valencia-California, USA), according to the manufacturer's instruction and the concentration of total RNA was determined by Nanodrop (Biotech, Vermont, USA). The cDNA was synthesized using 1 μg of total RNA samples according to the cDNA synthesis kit instruction (Fermentas, Frederick-Maryland, USA) and the resulting cDNA samples were subjected to PCR analysis using specific primer sets. PCR was carried out in tubes containing 1 μg of cDNA, 10 μl of SYBR Green master mix (Takara Bio USA Inc, California-USA), 1 μl of each of forward and reverse primers with 10 pM concentration, and 7 μl of deionized water to obtain final volume of 20 μl. Primers (Table 1) were designed by allele ID6.0 software based on the caspase-3 and *p38 MAPK* gene sequences indicated in the Gene Bank online resource (http://www.ncbi.nlm.nih.gov/). PCR amplification was carried out under following conditions: initial denaturation at 95 °C for 30 s, followed by 40 cycles of 95 °C for 5 s, annealing at 43.2 °C for caspase 3, 51.5 °C for *p38 MAPK*, or 53.2 °C for *18S rRNA*, and extension at 72 °C for 30 s, and a final extension at 72 °C for 10 min using a BioRad PCR thermocycler (BioRad, California, USA). 

**Table 1:** Target genes and their relative primer sequences

**Target gene**	**Primers**		**Amplicon size (bp)**
**Caspase-3**	Forward	5'-CAGCACCTGGTTATTATTCT-3'	98
	Reverse	5'-TTGTCGGCATACTGTTTC-3'	
***p38 MAPK***	Forward	5'- GCTGTGAATGAAGACTGTGAG-3'	165
	Reverse	5'- GCATCCCACTGACCAAATATC-3'	
***18S rRNA***	Forward	5'- GTAACCCGTTGAACCCCATT-3'	151
	Reverse	5'- CCATCCAATCGGTAGTAGCG-3'	

The validity of the observed signals from the RT-PCR were confirmed using melting peak and dissociation curve survey and by verifying the bands on 2% agarose gel electrophoresis, compared with the 100 bp DNA ladder marker. Relative expression of the studied genes was determined using 2^ΔΔCt^ which was normalized to *18S rRNA* reference gene expression (20). All experiments were conducted in duplicate and relative expression of genes was determined in comparison with the expression of *18S rRNA* gene as control.

**Flow cytometry for intracellular caspase-3 and p-p38 MAPK proteins assay**

To determine proapoptotic and antiprolif-erative effects of genistein on HT29 colon cancer cells at the protein level, an intracellular protein analysis was conducted using flow cytometry technique. Cellular levels of caspase-3 protein and phosphorylated p38 MAPK (p-p38 MAPK), the active form of p38 MAPK protein were determined in this experiment. The HT29 cells were seeded at a density of 110^4^ cells/ml in 75-cm^2^ flasks and treated with 30, 50, and 70 μM solutions of genistein for 48 h. Cells were then trypsinized, washed with PBS, fixed in 4% paraformaldehyde, and permeabilized on ice-cold methanol for 30 min. Next, cells were incubated for 60 min with primary antibody followed by 30 min incubation with secondary antibody for each corresponding protein (www.ptglab.com). Finally, cells were analyzed on a BD-FACScalibur flow cytometry instrument (BD Biosciences, California-USA). Mouse caspase-3 antibody (E-8), as primary antibody and donkey anti-mouse IgG-PE and mouse normal IgG2a-PE, as secondary and normal antibodies respectively were used for determination of intracellular caspase-3 protein. Similarly, rabbit phospho-p38 MAPK (Thr180/Tyr182) antibody was used as primary antibody whereas donkey anti-rabbit IgG-PE and rabbit normal IgG-PE were applied as secondary and normal antibodies respectively to determine p-p38 MAPK protein in HT29 cells. All antibodies were purchased from Santa Cruz (Santa Cruz Biotechnology, Inc, Heidelberg-Germany).

**Determination of the effect of genistein on caspase-3 enzyme activity**

Caspase-3 enzyme activity was determined using colorimetric caspase-3 assay kit (Abcam®, Cambridg, UK), according to the manufacturer's instructions. Briefly, 5×10^6^ cells were plated in 25 cm^2^ flasks and incubated with 0, 30, 50 or 70 µM genistein. The cells were harvested after 48 h and lysed in 50 μl of cold lysis buffer (1% of Triton-X100 and 0.015% of DTT). The cells were then centrifuged at 10,000×g for 1 min and supernatants were transferred to ice-cold fresh tubes for immediate assay. The assay was based on the spectrophotometric detection of chromophore p-nitroaniline (*p*-NA) after cleavage from the labeled substrate DEVD-*p*-NA. The *p*-NA light emission was quantified using a Sunrise^TM^ microtitre plate reader (Tecan Group Ltd, Männedorf-Switzerland) at 405 nm. Finally, The fold increase in caspase-3 activity was determined by comparison of the absorbance of *p*-NA from treated sample with that of control sample.

**Statistical analysis**

Data was analyzed by SPSS 16 (SPSS Inc., Chicago, USA) and One Way ANOVA followed by Tukey-Kramer multiple comparisons test to analyze the the differences between groups. For real-time PCR the difference in the initial concentration of each transcript, normalized with respect to control using comparative Ct method and ΔΔCt values were calculated. The changes in gene expressions were calculated using the formula: Fold Change= 2^-ΔΔCt^, where ΔΔCt= ΔCt for treatment- ΔCt for control and ΔCt is the difference between the Ct value of the target gene and the reference gene. Results were presented as meanSD and the p-value less than 0.05 was considered as significant.

## Results

**Genistein inhibited viability of HT29 cells**

**Fig. 1:**
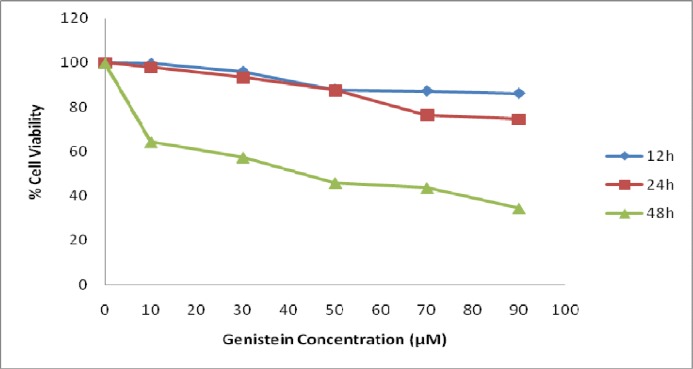
The effect of genistein on HT29 cell viability. Exposure of HT29 cells to different concentration of genistein for 12, 24, and 48 h decreased cell viability in a dose-dependent manner. The incubation of the cells with genistein for 48 h exhibited greater decline in the cell viability compared with shorter incubation time

Addition of genistein to HT29 cells decreased the percentage of viable cells after 48 h of incubation but not after incubation for 12 and 24 h, as shown in Figure 1. Cells were detached from culture flasks after incubation with genistein for more than 56 h making viable cell count inapplicable (data not shown). In contrast, 48 h of incubation with genistein significantly (P<0.01) inhibited the cell survival and decreased cell viability in a dose-dependent manner. The 50 μM concentration of genistein was found as IC_50_ of genistein for HT29 cells. However, 30, 50, and 70 μM genistein were also used in further experiments to confirm 50 μM as optimum concentration of Genistein.

**Genistein prevented migration of HT29 cells**

**Fig. 2:**
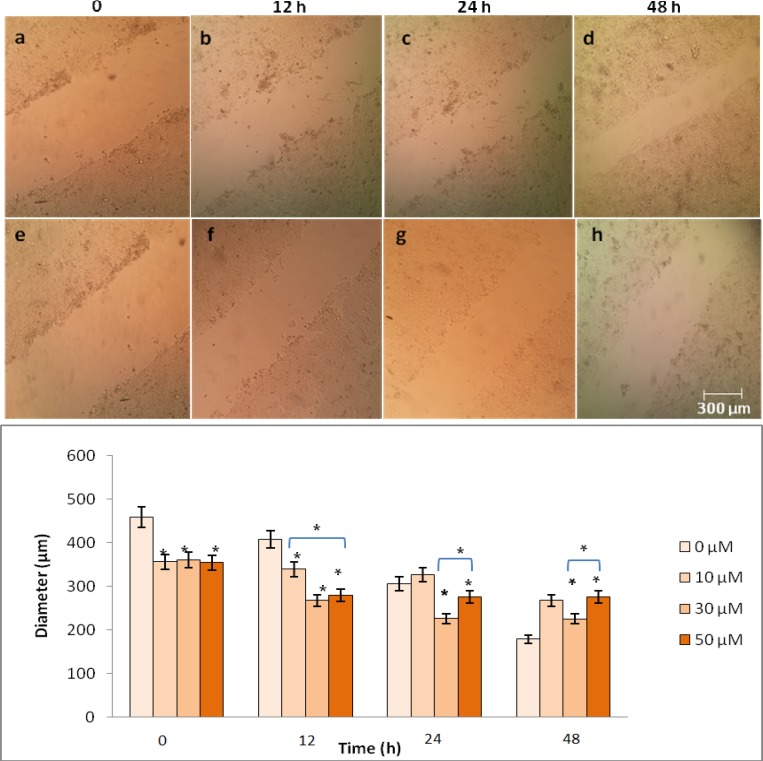
The effects of genistein on HT29 cell migration analyzed by wound healing assay. Cells were cultured with 0, 10, 30 and 50 µM genistein for 0, 12, 24 and 48 h, a wound track was scored and the number of migrated cells was determined. Top**:** the number of migrated cells was significantly reduced after exposure of HT29 cells to 50 µM genistein for 48 h (e-h) compared with untreated control group (a-d). Bottom: wound diameter (µm) was determined and One-Way-ANOVA analysis was performed to compare differences between groups. The symbol (*) represents significant differences (P< 0.05) between groups compared to untreated cells

An *in vitro* wound healing assay was performed to determine the effect of genistein on HT29 cell migration. As shown in Figure 2, the width of the gap between culture areas was decreased by the increasing migrated cells in the control group after 48 h. However, the number of migrated cells was significantly reduced following the incubation of HT29 cells with 50 µM genistein.

**Genistein inhibited proliferation of HT29 cells**

**Fig. 3:**
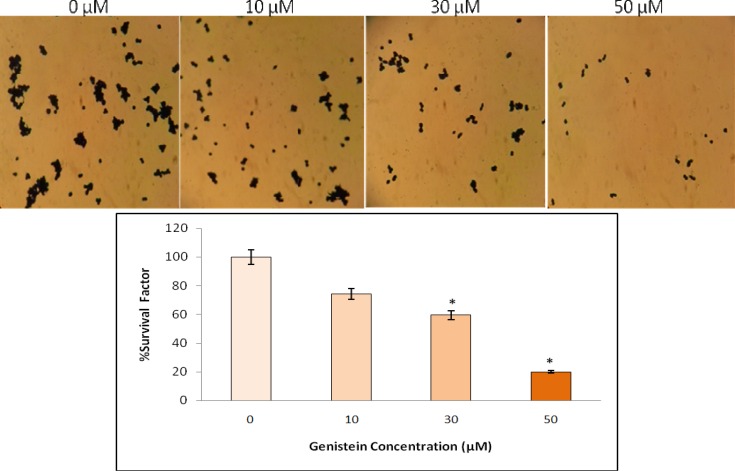
The effects of genistein on the proliferation of HT29 cells analyzed by colony formation assay. Cells were plated and treated with 0, 10, 30 and 50 µM genistein for one week. The number of colonies was determined, plating efficiency was calculated and the percentage of survival factor was obtained. Figure shows the stained cultured cells (top) and survival factor (%) based on different concentration of genistein (bottom). One-Way-ANOVA analysis was performed to compare the differences between groups and the symbol (*) represents significant differences (P< 0.05) between groups compared to untreated cells

An *in vitro* clonogenic assay showed that genistein significantly inhibited HT29 colon cancer cell proliferation in a dose-dependent manner. As shown in Fig. 3, treatment of HT29 cells with 30 and 50 µM genistein resulted in 40% and 80% reduction in proliferation respectively, indicating that the higher concentration of genistein induces the greater inhibition of cell proliferation.

**Genistein increased apoptosis and reduced proliferation of HT29 cells**

**Fig. 4:**
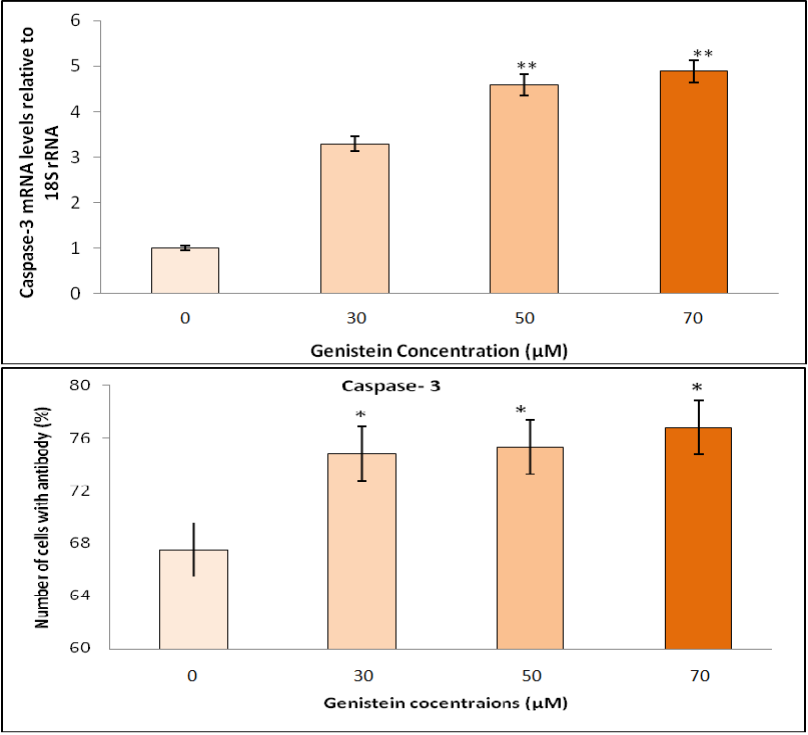
Dose-dependent proapoptotic effects of genistein on HT29 cells, as determined by RT-PCR and flow cytometry. The relative gene expression of caspase-3 in HT29 cells upon exposure to 0, 30, 50 and 70 µM genistein was examined by RT-PCR. Caspase-3 gene expression was increased by increasing genistein concentrations compared with *18S rRNA* gene expression (top). Genistein also induced significant apoptosis in HT29 cells by increasing caspase-3 protein compared to untreated cells, as determined by flow cytometry (bottom). One-Way-ANOVA analysis was performed to compare differences between groups. (*) and (**) indicate significant difference at P< 0.05 and P< 0.01 levels compared with untreated cells, respectively

**Fig. 5:**
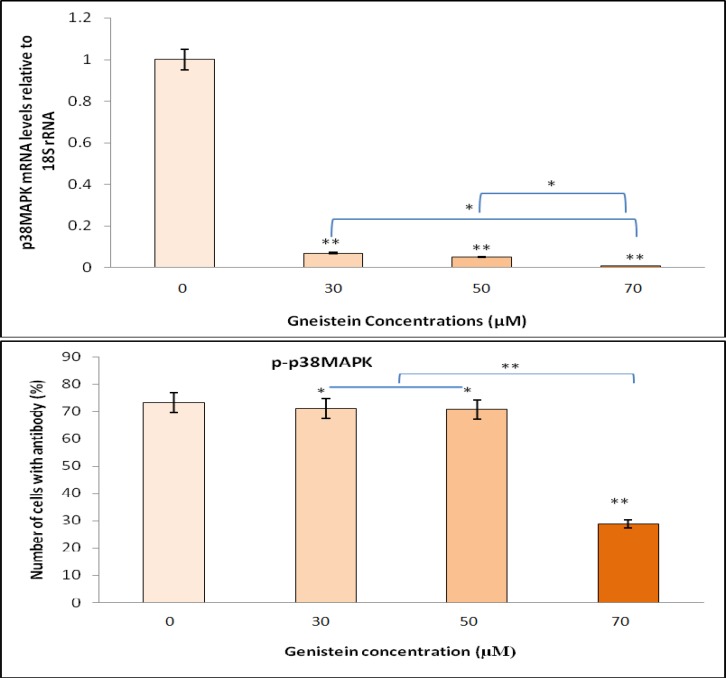
Dose-dependent antiproliferative effects of genistein on HT29 cells, as determined by RT-PCR and flow cytometry. The relative gene expression of *p38* *MAPK *in HT29 cells upon exposure to 0, 30, 50 and 70 µM genistein was examined by RT-PCR. *P38* *MAPK* gene expression was reduced by increasing genistein concentrations compared with *18S rRNA* gene expression (top). Genistein also significantly inhibited HT29 cells proliferation by reducing p-p38 MAPK protein compared to untreated cells, as determined by flow cytometry (bottom). One-Way-ANOVA analysis was performed to compare differences between groups. (*) and (**) indicate significant difference at P< 0.05 and P< 0.01 levels compared with untreated cells, respectively

To investigate the potential pro apoptotic property of genistein, HT29 cells were treated with 30, 50 or 70 μM genistein for 48 h and the expression of caspase-3 and *p38 MAPK *genes were determined. Genistein showed strong apoptotic activity by 3.29-, 4.59-, and 4.89-fold increase in caspase-3 gene expression corresponding to the exposure of HT29 colon cancer cells to 30, 50 and 70 μM genistein, respectively (Fig. 4). On the other hand, the relative expression of *p38 MAPK*, as a marker for proliferation was almost completely inhibited by 93, 95, and 97%, respectively after 48 h exposure to genistein (Fig. 5) compared with untreated cells (relative to the expression of *18S rRNA* housekeeping gene).

Proapoptotic and anti proliferative effects of genistein were investigated in HT29 cells at the protein level by flow cytometry using caspase 3 and p-p38 MAPK specific antibodies. HT29 cells were treated with different concentrations (30, 50 or 70 μM) of genistein for 48 h and the concentrations of caspase 3 and p-p38 MAPK proteins were measured. Flow cytometry showed a significant increase in caspase-3 protein level indicating that apoptosis markedly increased (P<0.05) in HT29 cells in a dose-dependent manner after exposure to genistein (Fig. 4). In addition, decreasing in p-p38 MAPK protein confirmed a significant dose-dependent decline (P<0.05) in cell proliferation (Fig. 5). Although both proapoptotic and antiproliferative effects of genistein were significantly detected in HT29 cells treated with 50 μM genistein, the greatest antiproliferative effect of genistein was observed with 70 μM genistein.

**Genistein reduced MMP2 and enhanced caspase-3 activity in HT29 cells**

**Fig. 6:**
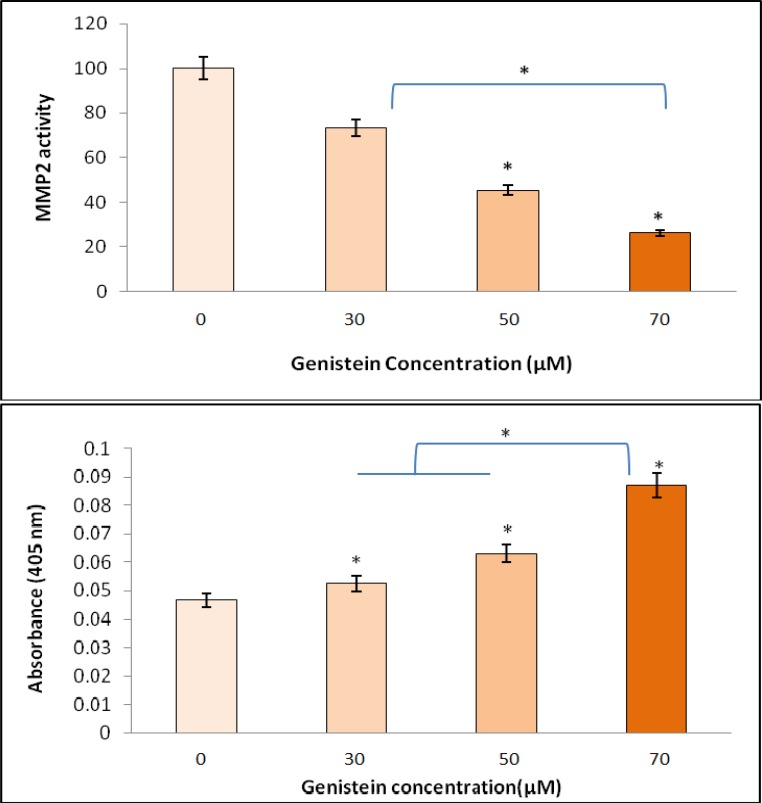
The effect of 0, 30, 50 and 70 µM genistein on MMP2 caspase-3 enzyme activities in HT29 cells. Top: cells were incubated for 48 h and conditioned media were used for the measurement of MMP2 activity by gelatin zymography. The enzyme activity (MMP2) was reduced by increasing genistein concentrations. Bottom**:** caspase-3 activity was significantly increased with increasing genistein concentrations and the greatest enhancement in caspase-3 activity (89%) was observed in the presence of 70 µM genistein. One-Way-ANOVA analysis was performed to compare differences between groups and the symbol (*) represents significant differences (P< 0.05) between groups compared to untreated cells

Metastasis contributes to tumor invasion by stimulating matrix metalloproteinase production. We used gelatin zymography to investigate the effect of genistein on HT29 cell MMP2 enzyme activity. As shown in Fig. 6, genistein significantly reduced the activity of MMP2 in HT29 cells in a dose-dependent manner (P<0.05). The exposure of the cells with 50 µM genistein significantly reduced MMP2 activity compared with untreated cells. However, the lowest MMP2 activity was observed after treatment of HT29 cells with 70 μM genistein confirming that the higher the concentration of genistein, the lower the activity of MMP2. 

On the other hand, HT29 colon cancer cells showed gradual increase in caspase-3 activity after exposure of the cells to 0,30,50, or 70 µM genistein (Fig. 6) with the highest activity being observed in 70 µM treated cells. Caspase-3 activity was increased 13, 36, and 89% in the presence of 30, 50, and 70 µM genistein, respectively.

## Discussion

The use of herbal therapy in different diseases is increasing worldwide and the effectiveness of herbal medicine as an adjunctive therapy have recently attracted considerable interest for their lipid lowering effects (21), antioxidant and antidiabetic effects (22), and anticancer properties (23). Polyphenolic and isoflavone components of plants are the major components of soy isoflavones. Genistein has clearly exhibited anticancer effects in a time-and dose-dependent manner, which might probably make it a competitive candidate for anticancer research (24). Regardless, the mechanism of its anticancer effects remains to be fully understood.

Considering the literature data, tumor cells show immortality in some degrees and do not respond to the cellular death signals and therefore do not normally undergo apoptosis. Also, tumor cells escape from cell cycle check points and proliferate uncontrollably, they migrate from original site of tumor and spread in the body to other areas (metastasis), and new capillary blood vessels may form (angiogenesis) to deliver oxygen and nutrients to growing tumor (25). Accordingly, specific characteristics and biological processes such as apoptosis, proliferation, migration (metasta-sis), and angiogenesis could be exploited as targets for the prevention and therapeutic goals of cancers. In this study, genistein was used as an important compound to delineate three proapoptotic, antipro-liferative, and anti migratory effects of soy iso-flavone on HT29 colon cancer cells. Our results showed that the soy isoflavone genistein strongly promoted apoptosis, remarkably reduced survival, and significantly inhibited migration and prolifera-tion of HT29 human colon cancer cells 

Apoptosis is characterized by a series of morphological alterations including plasma and nuclear membrane blebbing, cell shrinkage, dissolution of nuclear lamina, and the biochemical processes including activation of caspases (26). Apoptosis is controlled by two diverse pathways, the intrinsic or mitochondrial mediated pathway and the extrinsic or death receptor mediated pathway. The intrinsic pathway involves the releasing of cytochrome c from mitochondria and activation of caspase-9 and caspase-3 whereas the extrinsic pathway is initiated by the interaction of the ligand with its death receptor, which leads to the activation of caspase-8 and caspase-3. Therefore, caspase-3 has been known as a key mediator of apoptosis (27).

Interestingly, we showed that exposure of HT29 cells to 30, 50 and 70 µM solutions of genistein upregulated caspase-3 gene expression in a time- and dose-dependent manner. Based on this finding we hypothesized that genistein may induce apoptosis of HT29 colon tumor cells by increasing caspase-3 gene expression. We further confirmed this hypothesis by observing the increased intracellular concentration of caspase-3 protein in response to the exposure of HT29 cells to genistein. There is compelling evidence showing that apoptosis is mostly induced after exposure of the tumor cells to over 40-50 µM genistein (28). Likewise, 50 μM was found in this study as the optimum concentration of genistein for inhibiting viability, preventing proliferation, and reducing migration of HT29 cells, confirming that our observations are consistent with previous reports. Moreover, apart from the upregulation of caspase-3 gene expression and augmenting its protein level, genistein also enhanced the enzymatic activity of caspase-3 in HT29 cells which suggested a possible potency for genistein to boost the process of converting procaspase-3 to the active caspase-3 enzyme. The importance of caspase-3 activity in the induction of apoptosis in tumor cells has been the focus of several investigations (29, 30). According-ly, a simple electrochemical approach has recently been developed to determine caspase-3 activity as a key biomarker for apoptosis (31). Even so, as per our knowledge, there is no compelling evidence in the literature to show the effect of genistein on caspase-3 in the three gene expression, protein, and activity levels in cancerous cells, particularly in colon cancer cell lines. Our finding is of great importance since it is entirely examining the effect of genistein on caspase-3 involving pathways in HT29 cells. Altogether, these observations indica-ted a pivotal role for genistein in provoking apoptosis in cancerous cells.

The proapoptotic property of genistein, as we observed in this study is consistent with the results of previous studies. As reviewed in the literature by Mukhtar E et al. (2012) and Khan et al. (2010), exposure of the cells to the genistein induces apoptotic effects via different mechanisms, mainly by caspase activation, the activation of several endoplasmic reticulum stress regulators, and Bax/Bcl-2 ratio upturn (27, 32), although additional mechanisms have also been advanced, such as the inhibition of the proteasome activity (33) or the down regulation of antiapoptotic survivin (28). For instance, Park et al. have shown that exposure of anaplastic lymphoma cell line to the genistein induces changes in mitochondrial membrane potential and activates caspase-3 (34) and have concluded that upregulation of caspase-3 gene might be a candidate target for genistein-based treatment of cancer cells. Apart from the foster in the expression of caspase-3 gene, genistein has also been found to induce apoptosis by upregulating the expression of *BAX* and slightly decreasing BCL-2 level in cancerous cells (15). Yu et al. demonstrated that genistein inhibited the viability of HT29 human colon cancer cells by induction of apoptosis mainly through the regulation of the Bax/Bcl-2 expression ratio. Increased pro apoptotic caspase-3 protein level and enhanced Bax/Bcl-2 ratio in the presence of genistein have also been observed in MCF-7 human breast cancer cells (28). Considering these data, it can be concluded that genistein is able to evoke apoptosis in HT29 cells through mechanisms such as up regulation of the caspase-3 and therefore, may play a pivotal role in the induction of apoptosis and reduction of growth capacity in this cell line 

In the present study, in addition to confirming proapoptotic potency of genistein in HT29 colon cancer cells through the activation of caspase-3 involving pathways, we showed that genistein inhibited proliferation and reduced survival of cultured HT29 cells. Additionally, genistein strongly prevented the migration and invasion of HT29 cells. We believe that these observed inhibitory effects of genistein on proliferation and migration processes are mediated through MAPK pathway since the down regulation of *p38 MAPK* gene expression and the reduction of phosphrylated p38 MAPK (p-p38 MAPK) protein level as an active form of p38 MAPK protein, were also observed in the current study. In accordance with this notion, the importance of MAPK signaling pathway in the conversion of many extracellular stimuli into specific cellular responses such as cellular proliferation, differentiation, embryogen-esis, and cell death has recently been reported (35). As reviewed in the literature by Amado et al, MAPK pathway is a target for genistein whereby the modulation of MAPK by genistein promotes further indirect effects on nuclear factor kappa B (NF-B) pathway, enhancing the antitumor effects of this natural product by regulating various aspects of cellular growth, invasion, and angiogenesis (36). The inhibitory effects of genistein on NF-B pathway may also be mediated through cross-talk between Notch and NF-B signaling pathways processes via other pathways such as estrogen receptor signaling pathway (23).

It has convincingly been demonstrated that p38 MAPK pathway regulates colorectal cancer at different levels including tumor formation and metastasis (37). Metastatic cells exhibit over expression of matrix metalloproteinase enzymes that correlates with the attainment of their invasive potency. Interestingly, the expression of MMPs has already been shown to be mediated by p38 MAPK in bladder, breast, liver, skin keratinocytes, and prostate cancer cell lines (38). In line with these reports, we showed that genistein significantly reduced the activity of MMP2 in HT29 colon cancer cell line in a dose- dependent manner. Since p38 MAPK is necessary for transforming growth factor- (TGF-) mediated induction of MMP2 (39), the inhibitory effect of genistein on p38 MAPK gene expression and its phosphorylated active p-p38 MAPK protein as observed in this study, could indirectly block MMP2 activation and therefore inhibit cell metastasis. Therefore, our results together with these reports introduce MAPK signaling pathway as a target for genistein in cancer prevention and treatment .

Accumulating data have confirmed that many health benefits attributed to genistein have been observed at genistein concentration above 50 µM (23, 28). Consistently, we showed that IC_50_ for genistein is 50 µM and the highest apoptotic, anti proliferative, and anti metatstatic activity of genistein was observed at this concentration 

The limitations of this study should be considered when interpreting its findings. First, since the effects of genistein are partly mediated via NF-B and TGF-, therefore determination of these factors at the gene expression or protein level may strengthen the results. Second, animal models and *in vivo* investigations are required to validate *in vitro* observations. Finally, possible side effects of long term consumption of soy isoflavone genistein should be considered in cancer prevention and treatment.

Collectively, it can be concluded that genistein induces apoptosis in HT29 colon cancer cells by activating caspase-3 involving pathways, and inhibits proliferation and metastasis of the cells through blocking of p38 MAPK pathway at both *p38 MAPK* gene expression and protein levels. Therefore, the potential benefits of genistein in treatment and prevention of colon cancer might be the interest of further animal model studies and clinical trials. 
